# Summary of ‘The current status and future perspectives on the management of stage III NSCLC: a focus on unresectable cancer treatment paradigms’

**DOI:** 10.1038/s41416-020-01073-3

**Published:** 2020-12-08

**Authors:** Luis Paz-Ares, on behalf of AstraZeneca UK Limited

**Affiliations:** Department of Medical Oncology, Hospital Universitario 12 De Octubre, Madrid, Spain

Treatment decisions with curative intent for patients with stage III non-small cell lung cancer (NSCLC) often require a complex decision process, and benefit from continuous communication of expertise among, and between, lung cancer teams. In stage III NSCLC that is deemed unresectable, and in some resectable cases, the ongoing consensus is that concurrent chemoradiotherapy (cCRT) is the most appropriate treatment. Despite this, there are still low numbers of patients with stage III disease who undergo multi-modality cCRT treatment in the United Kingdom, in comparison with other parts of the Europe. The poor outcomes and lack of access to optimal treatment is a call to arms for UK lung cancer teams to improve patient outcomes, through prehabilitation and rehabilitation, to ensure that patients have the best physiological reserve for multi-modality treatment. An experienced multi-disciplinary team is required to assess patient eligibility and deliver cCRT to ensure maximum access for this potentially curative strategy.

Radiotherapy is a modulator of the immune response and tumour microenvironment; emerging evidence suggests that radiotherapy triggers the patients’ immune system to recognise the increase in T cell diversity. The synergistic effect between radiation and immune check point modulations has been demonstrated in multiple pre-clinical studies and has now been confirmed following the results of the Phase 3 PACIFIC trial, which demonstrated a significant improvement in progression-free survival and overall survival for adult patients with stage III unresectable NSCLC, whose tumours express PD-L1 on ≥1% of tumour cells, who received durvalumab (Imfinzi®**▼**; AstraZeneca UK Limited), when their disease had not progressed following the completion of platinum-based cCRT. Based on these results, durvalumab offers a standard of care as consolidation therapy for such patients. Pembrolizumab and nivolumab are approved for the treatment of patients with stage IV NSCLC and are being investigated in ongoing Phase II and III trials, in patients with stage III disease. Nivolumab was being investigated in a randomised controlled Phase III trial following cCRT (RTOG 3505; NCT02768558); however, this study was terminated in February 2019 because other treatments have been found to be efficacious, while pembrolizumab following cCRT demonstrated promising efficacy in an open-label Phase II trial in the same setting (LUN 14-179; NCT02343952).

In summary, the rapidly expanding role of immunotherapy for metastatic stage IV NSCLC has provided lung cancer teams with the expertise to apply their knowledge to curative stage III NSCLC, offering a more positive outlook for patients, and clinicians, with the introduction of durvalumab for eligible patients with stage III NSCLC.

## Appendix

**Prescribing information**

**IMFINZI**® **▼(durvalumab) 50** **mg/ml solution for infusion**

https://medicines.astrazeneca.co.uk/content/dam/multibrand/uk/en/prescribinginformation/imfinzi-pi.pdf

